# Diaphragmatic ultrasonography in patients with IPF: Is diaphragmatic structure and mobility related to fibrosis severity and pulmonary functional changes?

**DOI:** 10.5578/tt.20239903

**Published:** 2023-03-10

**Authors:** G. Kalbaran Kısmet, O. Okutan, Ö. Ayten, C. Samancı, M. Yeşildal, Z. KARTALOĞLU

**Affiliations:** 1 Clinic of Pulmonary Medicine, Sultan 2. Abdülhamid Han Training and Research Hospital, İstanbul, Türkiye; 2 Department of Radiology, İstanbul Cerrahpaşa University Faculty of Medicine, İstanbul, Türkiye; 3 Clinic of Radiology, Sultan 2. Abdülhamid Han Training and Research Hospital, İstanbul, Türkiye

**Keywords:** diaphragmatic ultrasonography, idiopathic pulmonary fibrosis, pulmonary function tests, high resolution computed tomography, fibrosis score

## Abstract

**ABSTRACT:**

Diaphragmatic ultrasonography in patients with IPF: Is diaphragmatic structure and mobility related to fibrosis severity and pulmonary functional changes?

**Introduction:**

There is evidence to suggest that dyspnea and impaired exercise capacity are associated with respiratory muscle dysfunction in idiopathic pulmonary fibrosis (IPF) patients. We aimed to evaluate the functions of the diaphragm with ultrasonography (US) and to determine the correlation of the data obtained with the pulmonary function parameters of the patients, exercise capacity, and the extent of fibrosis radiologically.

**Materials and Methods:**

Diaphragmatic mobility, thickness, and thickening fraction (TF) were measured by ultrasonography in IPF patients and the control group. The correlation between these measurements, pulmonary function tests (PFT), six-minute walking test (6MWT), mMRC score, and total fibrosis score (TFS) was evaluated.

**Results:**

Forty-one IPF patients and twenty-one healthy volunteers were included in the study. No difference was found between the patient and control groups in diaphragmatic mobility during quiet breathing (QB) on ultrasound (2.35 cm and 2.56 cm; p= 0.29). Diaphragmatic mobility during deep breathing (DB) was found to be lower in the patient group when compared to the control group (5.02 cm and 7.66 cm; p< 0.0001). Diaphragmatic thickness was found to be higher during QB and DB in IPF patients (0.33 cm and 0.31 cm, p= 0.043; 0.24 cm and 0.22 cm, p= 0.045). No difference was found between the two groups in terms of thickening fraction (39.37%, 44.16%; p= 0.49). No significant correlation was found between US measurements and PFT, 6MWT, mMRC score, and TFS in IPF patients (p> 0.05).

**Conclusion:**

The functions of the diaphragm do not appear to be affected in patients with mild-to-moderate restrictive IPF. This study showed that there was no relationship between diaphragmatic functions and respiratory function parameters and the extent of fibrosis. Further studies, including advanced stages of the disease, are needed to understand the changes in diaphragmatic functions in IPF and to determine whether this change is associated with respiratory function parameters and the extent of fibrosis.

## Abstract (tr)

**ÖZ:**

Noninvaziv solunum desteğinde akciğer ve diyafram ultrasonu: Gerçek bir araç mı yoksa moda mı?

**Giriş:**

İdiyopatik pulmoner fibrozis (İPF) hastalarında dispne ve bozulmuş egzersiz kapasitesinin solunum kas disfonksiyonu ile ilişkili olduğunu gösteren kanıtlar vardır. Diyafragmanın fonksiyonlarını ultrasonografi (US) ile değerlendirmeyi ve buradan elde edilen verilerin hastaların solunum fonksiyon parametreleri, egzersiz kapasitesi ve radyolojik olarak fibrozisin yaygınlığı ile korelasyonunu belirlemeyi amaçladık.

**Materyal ve Metod:**

İdiyopatik pulmoner fibrozis hastalarında ve kontrol grubunda diyafragma hareketi, kalınlığı ve kalınlaşma fraksiyonu (TF) US ile ölçüldü. Bu ölçümlerin solunum fonksiyon testleri (SFT), altı dakika yürüme testi (6DYT), mMRC skoru ve total fibrozis skoru (TFS) ile korelasyonu değerlendirildi.

**Bulgular:**

Kırk bir İPF ve yirmi bir sağlıklı gönüllü çalışmaya dahil edildi. Ultrasonografide sessiz solunum (QB) sırasında diyafragma hareketliliği açısından hasta ve kontrol grupları arasında fark bulunmadı (2,35 cm ve 2,56 cm; p= 0,29). Derin nefes alma (DB) sırasındaki diyafragma hareketliliği hasta grubunda kontrol grubuna göre daha düşük bulundu (5,02 cm ve 7,66 cm; p< 0,0001). Diyafragma kalınlığı İPF hastalarında QB ve DB sırasında daha yüksek bulundu (0,33 cm ve 0,31 cm, p= 0,043; 0,24 cm ve 0,22 cm, p= 0,045). Kalınlaşma fraksiyonu açısından iki grup arasında fark bulunmadı (%39,37, %44,16; p= 0,49). İdiyopatik pulmoner fibrozis hastalarında US ölçümleri ile SFT, 6DYT, mMRC skoru ve TFS arasında anlamlı bir ilişki bulunmadı (p> 0,05).

**Sonuç:**

Hafif-orta derecede restriksiyonu olan İPF hastalarında diyafragmanın fonksiyonları henüz etkilenmemiş gibi görünmektedir. Bu çalışma diyafragma fonksiyonları ile solunum fonksiyon parametreleri ve fibrozisin yaygınlığı arasında bir ilişki olmadığını göstermiştir. İdiyopatik pulmoner fibroziste diyafragma fonksiyonlarındaki değişiklikleri anlamak ve bu değişikliğin solunumsal fonksiyonel parametreler ve fibrozisin yaygınlığı ile ilişkili olduğunu belirlemek için hastalığın ileri evrelerini de içeren daha fazla çalışmaya ihtiyaç vardır.

## Introduction

Idiopathic pulmonary fibrosis (IPF), which constitutes a major group among idiopathic interstitial pneumonias, is a disease of unknown cause, limited to the lungs, characterized by progressive fibrosis and worsening of respiratory functions ( 1 , 2 ).

In interstitial lung disease (ILD) the increased elastic recoil places a significant burden on the diaphragm, which is the major respiratory muscle ( 3 , 4 ). In addition, chronic hypoxemia, corticosteroid usage, physical inactivity, malnutrition, systemic inflammation, and oxidative stress also cause muscle dysfunction (3,5-7). It has been shown that dyspnea and impaired exercise capacity in IPF are associated with respiratory muscle dysfunction, especially diaphragmatic dysfunction ( 8 , 9 ).

Changes in functional parameters in IPF are important in evaluating the severity of the disease, prognosis, and response to treatment. Recently, studies have been published on the evaluation of respiratory muscles with ultrasonography (US) and associating them with other functional measurements. Ultrasonography revealed decreased diaphragmatic mobility during deep breathing in ILD, which was associated with decreased lung volumes ( 10 , 11 ).

In our study, in IPF patients we evaluated the diaphragmatic function of the diaphragm, which is the major respiratory muscle, by assessing its thickness, mobility, and thickening fraction on US. The relationship between the data obtained here as well as the total fibrosis score (TFS), respiratory functions, and exercise capacity of the patients was investigated.

## MATERIALS and METHODS

### Study Population

Forty-one stable IPF patients, who were diagnosed according to the ATS/ERS 2011 and/or 2018 IPF Diagnostic Guidelines and followed up in the University of Health Sciences Sultan 2. Abdülhamid Han Training and Research Hospital Chest Diseases Polyclinic between January 2016 and February 2021, and 21 healthy volunteers with similar demographic characteristics to the patient group were included in this study.

The Turkish Ministry of Health Haydarpaşa Numune Training and Research Hospital Clinical Research Ethics Committee granted approval on March 23, 2020, with the decision number 2020/34. This study was conducted in accordance with the Good Clinical Practice Guidelines and the Declaration of Helsinki.

Written informed consent was obtained from all patients and volunteers participating in the study.

### Study Inclusion Criteria

• Idiopathic pulmonary fibrosis patients over 18 years of age with a BMI between 18.5 and ≤30 kg/m2, who were diagnosed according to the ATS/ERS 2011 and 2018 IPF guidelines were included.

### Study Inclusion Criteria

• Patients with chronic obstructive pulmonary disease (COPD), combined pulmonary fibrosis and emphysema (CPFE), using systemic corticosteroids, a history of pleural disease, and a history of upper abdomen and thoracic surgery were not included.

### Study Inclusion Criteria

The following were recorded for all the participants included in our study:

• Demographic characteristics: age, gender, BMI (kg/m2)

• Smoking history (pack/year, active smoker, former smoker, never smoked)

• Symptoms (dyspnea, cough)

• PFT, DLCO

• 6MWT (in the patient group only)

• Thickening fraction (TF)= [(Tmax - Tmin)/Tmin] x 100

• TFS in high-resolution computed tomography (HRCT)

Dyspnea scores were recorded as mMRC score (modified medical research council dyspnea scale). The six-minute walk test was performed according to standard criteria in the 2018 ATS guideline ( 12 ). Diaphragmatic US was performed on the same day as pulmonary function tests in all patients and the control group.

### PFT & DLCO

PFT measurement was performed with WinspiroPRO computer-based spirometry (MiniSpir, Rome, Italy) at least three times while the subjects were at rest, and the best result was evaluated and FVC values were recorded. SFT and DLCO were measured according to ATS/ERS standards (Viasys VMAX Encore, Germany) ( 13 , 14 ).

### Diaphragmatic Ultrasonography

**Figure 1:**
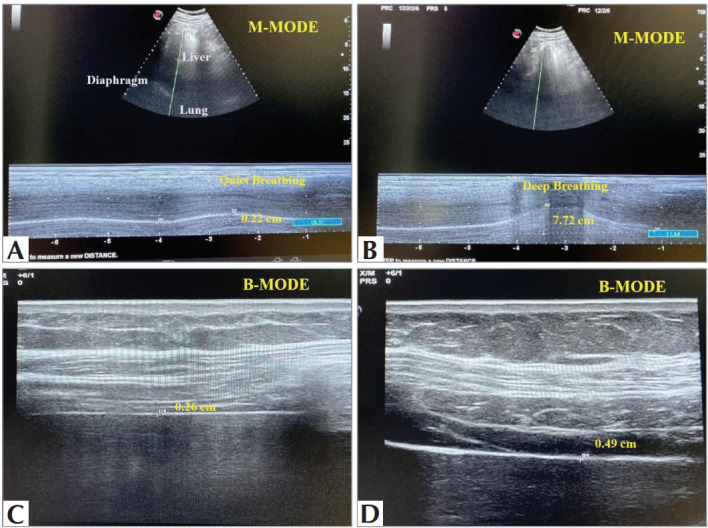
A. Diaphragmatic excursion was measured by freezing the image of the diaphragmatic curve during quiet breathing and centering the distance between echogenic lines, B. Diaphragmatic excursion was measured by freezing the image of the diaphragmatic curve during deep breathing and centering the distance between echogenic lines, C. Diaphragmatic thickness was measured during quiet breathing (QB) on functional residual capacity (FRC), D. Diaphragmatic thickness was measured during maximal deep breathing (DB) on total lung capacity (TLC).

Ultrasonography of the right diaphragm (Esaote, MyLab, Genoa, Italy) was performed while the patients were in the supine position. All participants underwent imaging using a low anterior subcostal, coronal intercostal approach, or both. After obtaining the best view in two-dimensional mode, diaphragmatic mobility (excursion) was measured in M mode. For the evaluation of diaphragmatic excursion, a 1-8 MHz convex transducer was placed on the anterior subcostal region between the midclavicular and anterior axillary lines. Diaphragmatic excursion was measured by freezing the image of the diaphragmatic curve during quiet ( Figure 1A ) and deep breathing ( Figure 1B ) and centering the distance between echogenic lines. For US image recording during deep breathing, all participants were instructed to exert maximum inspiratory effort for at least two seconds to achieve a maximum lung volume close to TLC ( 10 ).

Diaphragmatic thickness was measured in B mode US with a 3-13 MHz linear transducer placed between the right anterior and mid-axillary lines, next to the costophrenic angle on the diaphragmatic localization region. Thickness was measured from the most superficial pleural line to the deepest peritoneal line. Diaphragmatic thickness was measured first during quiet breathing (QB) on functional residual capacity (FRC) ( Figure 1C ), then during maximal deep breathing (DB) on total lung capacity (TLC) ( Figure 1D ). The diaphragmatic thickness measured during quiet breathing was recorded as Tmin, and the diaphragmatic thickness measured during deep breathing was recorded as Tmax in cm.

Diaphragmatic thickening fraction (TF) was calculated with the formula TF= [(Tmax - Tmin)/Tmin] x 100 ( 10 ).

Measurements in the patients and control groups were made by the same radiologists experienced in thoracic radiology. The average of the three most effective measurements, as well as the average of the measurements of the two radiologists, were recorded in centimeters. The radiologists were not informed about the health status of the participants.

### Calculation of Fibrosis Score on High-Resolution Computed Tomography

Six anatomically defined axial sections were selected for analysis. Traction bronchiectasis (TB), reticulation (R), honeycomb (H), and traction bronchiectasis with ground glass opacity (TB + GGO) HRCT abnormalities defined according to Fleischner-based nomenclature in each section for both lungs were expressed between 0 and 100 as percent (%). Pure ground glass areas without traction bronchiectasis were not included in the scoring. Total fibrosis score (TFS) was calculated by summing the H, R, TB, TB + GGO scores of each section ( 15 ). The mean value of the calculations of the two radiologists separately was accepted as the TFS value.

Fibrosis scores were calculated by two different radiologists experienced in thoracic radiology from the current HRCTs of the patients. The average of the calculations of the two radiologists separately was accepted as the TFS value.

### Statistical Analysis

The study included 62 participants. Parametric tests were used without the normality test due to the compatibility of the central limit theorem ( 16 ). In the analysis of the data, when performing statistics for continuous data on scales the mean, median and standard deviation, minimum and maximum values of the characteristics were used; frequency and percentage values were used to define categorical variables. Student’s t-test was used to compare the means of two groups in continuous measurements.

Chi-square test and Cochran-Armitage statistics were used to evaluate the relationship between categorical variables. Pearson correlation or Spearman correlation coefficient was used when examining the relationships between continuous measurements. Roc curve analysis was used to determine the cut-off values of the parameters.

The statistical significance level of the data was taken as p< 0.05. In the evaluation of the data, www.e-picos.com New York software and MedCalc statistical package program were used.

**Table 1:** Demographic and clinical characteristics of the IPF patients and control group are significant at p< 0.05 (*Student’s t/**Chi-square)

		IPF group (n= 41)	Control group (n= 21)	
Descriptive characteristics		x̄ ± SD	x̄ ± SD	p
Age		72.1 ± 7.3	71.1 ± 6.9	0.61*
		n (%)	n (%)	p
Gender	Male	25 (61)	12 (57.1)	0.77**
	Female	16 (39)	9 (42.9)	
Dyspnea	No	4 (9.8)	-	-
	Yes	37 (90.2)	-	-
Cough	No	8 (19.5)	-	-
	Yes	33 (80.5)	-	-
mMRC score	0	4 (9.8)	-	-
	1	11 (26.8)	-	-
	2	13 (31.7)	-	-
	3	11 (26.8)	-	-
	4	2 (4.9)	-	-
Smoking status	Never smoked	16 (39)	9 (42.9)	0.58**
	Active smoker	3 (7.3)	3 (14.3)	0.58**
	Former smoker	22 (53.7)	9 (42.9)	0.58**
SpO_2_ change	No	10 (24.4)	-	-
	Yes	31 (75.6)	-	-
SpO_2_ change level		5.22 ± 2.95	-	-
6MWD (m)		415.46 ± 100.32	-	-
BMI (kg/m^2^)		26.68 ± 2.47	26.21 ± 2.7	0.49*
Smoking (pack/year)		37.88 ± 23.43	25.61 ± 24.21	0.14*
DLCO (%)		58.95 ± 19.61	94.04 ± 15.33	<0.001*
FVC (%)		78.97 ± 18.68	108.19 ± 23.36	<0.001*

SD: Standard deviation, mMRC: Modified medical research council, SpO2: Peripheral oxygen saturation, 6MWD: Six-minute walk distance, BMI: Body mass index, DLCO: Diffusing capacity for carbon monoxide, FVC: Forced vital capacity, IPF: Idiopathic pulmonary fibrosis.

**Table 2:** Ultrasonographic measurements of the IPF patients and control group are significant at p< 0.05 (*Student’s t)

	IPF group (n= 41)	Control group (n= 21)	
Variables	x̄ ± SD	x̄ ± SD	p
QB diaphragmatic thickness (cm)	0.24 ± 0.05	0.22 ± 0.04	0.045*
DB diaphragmatic thickness (cm)	0.33 ± 0.05	0.31 ± 0.04	0.043*
QB diaphragmatic excursion (cm)	2.35 ± 0.77	2.56 ± 0.66	0.29*
QB diaphragmatic thickness (cm)	5.02 ± 1.57	7.66 ± 0.93	<0.001*
(95% CI)	(4.54-5.5)	(7.26-8.06)	<0.001*
TF %	39.37 ± 22.81	44.16 ± 31.41	0.49*
median (min-max)	38.46 (5.88-100)	40.9 (6.45-141.17)	0.49*

QB: Quiet breathing, DB: Deep breathing, TF: Thickening fraction, IPF: Idiopathic pulmonary fibrosis.

## RESULTS

Forty-one patients with IPF and 21 healthy subjects were included in our study. There was no difference between the control group and the patient group in terms of age, gender, BMI, and smoking status (p> 0.05) ( Table 1 ). The data of the patient and control groups are presented in Table 1 .

**Figure 2:**
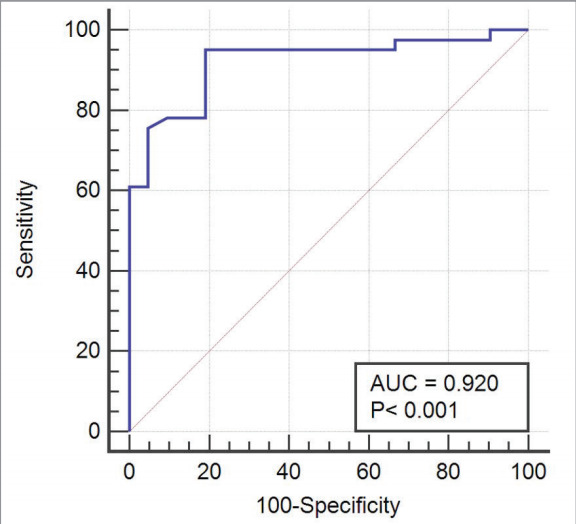
Specificity and sensitivity of the cut-off value (≤6.9 cm) for DB diaphragmatic excur.

No difference was found between the patient and control groups in diaphragmatic mobility during quiet breathing on US (2.35 cm and 2.56 cm; p= 0.29). However, diaphragmatic mobility during deep breathing was found to be lower in the patient group (7.66 cm and 5.02 cm; p< 0.0001) ( Table 2 ).Diaphragmatic excursion of ≤6.9 cm on US during deep breathing had a specificity of 99% and a sensitivity of 60.98% for decreased mobility [area under the curve (AUC): 0.920; p< 0.001] ( Figure 2 ).

Diaphragmatic thickness was found higher during quiet and deep breathing in IPF patients (0.24 cm and 0.22 cm in the healthy group, p= 0.045; 0.33 cm and 0.31 cm in the patient group, p= 0.043). No difference was found between the two groups in terms of TF (%) (39.37% vs. 44.16%; p= 0.49) ( Table 2 ).

No statistically significant relationship was found between US measurements (diaphragmatic mobility, thickness, TF) and functional parameters (FVC, DLCO, 6MWD, SpO2 change level), mMRC score, and TFS in IPF patients (p> 0.05) ( Table 3 ).

The mean TFS in IPF patients was 344.61 ± 167.82. The median value of TFS in the patients was 320. According to this value, IPF patients were divided into two groups, those with a high fibrosis score and a low fibrosis score ( Table 4 ). There was no difference between US measurements between these two groups.

In terms of US findings in IPF patients, no difference was found between the groups with high (mMRC≥ 2)/ low (mMRC< 2) dyspnea scores (p> 0.05).

## DISCUSSION

Changes in the lung parenchyma in ILD affect respiratory muscles as well as functional parameters. There is an increase in research on the evaluation of respiratory muscles assessed with US and their relationship to functional measurements.

In this study, in moderate-mild cases with a diagnosis of IPF and an FVC value above 50%; mobility and thickness of the diaphragm were measured with US, and the thickening fraction was calculated. The data obtained were compared with the patient and control groups. In addition, the correlation of US findings with functional measurements (DLCO, 6MWT, FVC, mMRC) and TFS values was evaluated.

**Table 3:** The Correlation between US Findings and physiological parameters is significant at *p< 0.05 (*Pearson/**Spearman)

		QB diaphragmatic thickness	DB diaphragmatic thickness	QB diaphragmatic excursion	DB diaphragmatic excursion	TF
TFS	r	-0.16	-0.03	0.12	0.13	-0.08
	p	0.31	0.85	0.46	0.41	0.63
FVC (%)	r	-0.23	-0.19	-0.19	0.1	-0.09
	p	0.15	0.24	0.23	0.51	0.56
DLCO (%)	r	-0.16	0.04	-0.29	0.27	-0.04
	p	0.32	0.8	0.06	0.09	0.78
mMRC score	r	-0.05	-0.03	0.28	0.02	0.24
	p	0.76	0.83	0.07	0.9	0.13
6MWD (m)	r	-0.2	-0.35	-0.07	-0.04	-0.19
	p	0.19	0.02	0.64	0.79	0.3
SpO_2_ change level	r	-0.12	-0.3	0.009	-0.19	-0.04
	p	0.53	0.1	0.96	0.31	0.78
BMI (kg/m^2^)	r	0.07	0.09	-0.2	-0.009	-0.24
	p	0.63	0.58	0.2	0.96	0.25
Smoking (pack/year)	r	-0.3	-0.22	-0.09	0.24	0.008
	p	0.14	0.3	0.68	0.25	0.96
Duration of treatment/disease (months)	r	-0.3	-0.22	0.12	0.12	-0.14
	p	0.06	0.16	0.46	0.46	0.38
Age (years)	r	-0.14	-0.21	0.08	-0.04	0.07
	p	0.37	0.19	0.61	0.81	0.68

QB: Quiet breathing, DB: Deep breathing, mMRC: Modified medical research council, SpO2: Peripheral oxygen saturation, 6MWD: Six-minute walk distance, BMI: Body mass index, DLCO: Diffusing capacity for carbon monoxide, FVC: Forced vital capacity, IPF: Idiopathic pulmonary fibrosis, TFS: Total fibrosis score, TF: Thickening fraction.

In patients with IPF, the diaphragmatic thickness was increased during quiet and deep breathing, and diaphragmatic mobility was decreased during deep breathing in US. No relationship was found between the data showing the diaphragmatic function measured with US and the patient’s FVC, DLCO, 6MWD, SpO2 change, and mMRC score. In addition, no significant relationship was found between TFS, which shows the radiological extent of the disease, and measurements obtained from US.

In our study, we found diaphragmatic dysfunction in patients with IPF, which was characterized by decreased diaphragmatic mobility during deep breathing on US. Santana et al. discovered a decrease in diaphragmatic mobility during deep breathing in ILD in two separate trials comparing patients with ILD and the healthy population ( 10 , 11 ). In another study by Boccatanda et al., in which 12 patients with IPF and 12 healthy patients were compared, it was reported that there was a decrease in diaphragmatic mobility in IPF patients during deep breathing ( 17 ). They explained that the absence of difference in diaphragmatic excursion during quiet breathing in ILD is due to the fact that, while the compliance of the fibrotic lung diminishes during rest, it maintains its current volume and hence moves within the limits during quiet breathing ( 17 ). Our findings are similar to the findings of these three studies. Our IFP patients were moderate and mild cases with an FVC value above 50%. As a result, we believe that there is no difference in diaphragmatic excursion during quiet breathing. Diaphragmatic excursion was found to be comparable to the healthy population in a study of patients with fibrosing alveolitis ( 18 ). On the other hand, He et al. examined the diaphragmatic excursion of 124 patients via ultrasonography, 18 of whom had IPF, and they found no difference in the diaphragmatic excursion between IPF and the healthy group during quiet and deep breathing ( 19 ). Also in this study, the FVC values of IPF patients had a mean of 70.18%. They found the lowest diaphragmatic mobility during deep breathing in the CPFE group among the healthy, IPF, CPFE, and COPD groups ( 20 ). They stated that IPF patients had near-normal diaphragmatic excursions since low lung volumes cause elongation in the diaphragm and sharpen the diaphragmatic curve in IPF patients ( 19 , 20 , 21 ).

**Table 4:** Statistics on the correlation/difference between values and TFS in IPF patients are significant at p< 0.05 (*Student’s t/**Cochran)

TFS		≤320 (n= 22)	>320 (n= 19)	
TFS		n (%)	n (%)	p
mMRC score	0	3 (13.6)	1 (5.3)	0.04**
	1	7 (31.8)	4 (21.1)	
	2	8 (36.4)	5 (26.3)	
	3	4 (18.2)	7 (36.8)	
	4	-	2 (10.5)	
		x̄± SD	x̄± SD	
Smoking (packs/year)		33.69 ± 25.18	42.42 ± 21.52	0.36*
BMI (kg/m^2^)		26.58 ± 2.38	26.8 ± 2.64	0.78*
FVC (%)		78.59 ± 20.59	79.42 ± 16.75	0.89*
DLCO (%)		68.81 ± 20.18	47.53 ± 11.11	<0.001*
6MWD (m)		440.86 ± 96.31	386.05 ± 99.19	0.08*
SpO_2_ change level		4.31 ± 2.65	6.2 ± 3.03	0.07*
QB diaphragmatic thickness (cm)		0.25 ± 0.06	0.24 ± 0.04	0.54*
DB diaphragmatic thickness (cm)		0.34 ± 0.06	0.33 ± 0.05	0.81*
QB diaphragmatic excursion (cm)		2.26 ± 0.84	2.45 ± 0.68	0.44*
DB diaphragmatic excursion (cm)		4.96 ± 1.66	5.08 ± 1.5	0.81*
TF (%)		38.77 ± 26.72	40.06 ± 17.96	0.86*

QB: Quiet breathing, DB: Deep breathing, mMRC: Modified medical research council, SpO2: Peripheral oxygen saturation, 6MWD: Six-minute walk distance, BMI: Body mass index, DLCO: Diffusing capacity for carbon monoxide, FVC: Forced vital capacity, IPF: Idiopathic pulmonary fibrosis, TFS: Total fibrosis score, TF: Thickening fraction, SD: Standard deviation.

These studies mostly included patients with non-IPF ILDs. Furthermore, patients with CTD-ILD who received steroid therapy were included, both of which may have an effect on the respiratory muscles. In addition, there are differences between studies in terms of age and functionality in patients. There have been few studies that specifically investigated diaphragmatic functioning in IPF patients using US. The number of patients in these studies was also limited.

The other parameter we evaluated with ultrasonography was the thickness of the diaphragm. We found diaphragmatic thickness to be higher during quiet and deep breathing in IPF patients than in healthy patients. In two studies assessing diaphragmatic functions with US in ILD, Santana et al. discovered that diaphragmatic thicknesses were thicker exclusively during quiet breathing in ILD compared to the healthy population ( 10 , 11 ). The thicker diaphragm during quiet breathing is believed to develop as a response to respiratory muscle overload ( 11 ).

Diaphragmatic thickness measured with US actually refers to indirect muscle mass and is affected by parameters such as BMI and distribution of muscle fibers. The thickening fraction, which reflects the size and function of the diaphragm, gives more accurate results ( 20 , 21 , 22 ). In the consensus report of ATS/ERS in 2019, the TF value for diaphragmatic dysfunction in the healthy population was expressed as less than 20% ( 22 ).

We found that diaphragmatic thickness increased during quiet and deep breathing in the IPF patient group. However, we discovered a similar mean TF of 39.37 percent in the IPF patient group and 40.99 percent in the healthy group. Santana et al., on the other hand, discovered low TF means in the ILD group compared to the healthy group in two separate studies (62% and 70%, respectively). In these studies, the diaphragmatic thickness was found to be decreased in ILD during deep breathing and therefore TF was detected to be low. They believed that this was because the muscle during quiet breathing was thick but dysfunctional, i.e. pseudohypertrophic, therefore  unable to thicken during deep breathing ( 11 ). They argued that low TF is indicative of diaphragmatic dysfunction ( 10 , 11 ).

In accordance with the 2019 ATS/ERS guideline, the TF values discovered in our and Santana’s research are within the normal range. We believe that the differences in TF levels between these studies are a result of the patient population characteristics.

The majority of studies evaluating US findings and functional measurements were performed with healthy populations. Cardenas et al. found a positive correlation between diaphragmatic thickness during deep breathing and FVC in healthy population ( 23 ). Boussuges et al. studied 210 healthy subjects, and found a weak positive correlation (p= 0.04) between diaphragmatic mobility and lung volumes (FVC, RV, FRC, TLC, FEV1/FVC) ( 24 ). Many similar studies showed a positive correlation between diaphragmatic excursion during deep breathing and FVC, and FEV1 ( 10 , 11 , 23 , 25 , 26 ).

In patients with ILD, Santana et al. found that decreased diaphragmatic motion during deep breathing was correlated with an increase in mMRC score, a decrease in 6MWD and an increase in SpO2 change, and a decrease in DLCO ( 10 ). They argued that excessive load on the inspiratory muscles in patients with ILD with decreased diaphragmatic mobility in deep breathing and reduced TF causes earlier dyspnea in patients. They also found a positive correlation between diaphragmatic excursion during deep breathing and FVC. Researchers state that an FVC below 60% is a good predictor of decreased diaphragmatic mobility in ILD ( 10 , 11 ).

In the two ILD studies conducted by Santana et al., the mean age of the patients was 49 and 55 years, and the mean FVC was 57% and 58%. Boccatonda et al. found a weak positive correlation between diaphragmatic excursion during both quiet and deep breathing and FVC in 12 patients with IPF with a mean age of 71 years and a mean FVC of 72% ( 17 ). Although the mean age of our IPF patient group was 72, the mean FVC was around 78%. Although we found a decrease in diaphragmatic mobility during deep breathing in IPF patients, we could not find a significant correlation with FVC, DLCO, 6MWD, SpO2 change, and mMRC score. Differences in the degree of restriction may account for these differences in correlations.

In our study, no relationship was found between diaphragmatic thickness and excursion, and gender and age in the IPF group and the healthy group. Likewise, Testa et al. also found no difference in diaphragmatic excursion during deep breathing in terms of age and gender ( 27 ). In the study of Cardenas et al., in the healthy population, diaphragmatic excursion and thickness were not affected by gender and age during quiet breathing, while diaphragmatic excursion and thickness were found to be lower during deep breathing in the elderly and women ( 24 ).

We did not find a significant relationship between TF value and gender, age, functional measures, and TFS in patients with IPF. Cardenas et al. found lower TF values in females than males in the healthy population. They also found a positive correlation between TF and age, FVC, and inspiratory muscle strength ( 23 ). Santana et al. found a decrease in TF in the ILD group but found no correlation between FVC, DLCO, TLC, and mMRC score ( 11 ). In the researcher’s other study involving the ILD group, TF was found to be correlated with an increase in dyspnea (mMRC score), a decrease in exercise tolerance (6MWD, SpO2 change), and poor lung functions (FVC, DLCO) ( 10 ). These studies include a small number of cases with different demographic characteristics. Likewise, ILD has a wide spectrum, and fibrosis rates show differences. Therefore, it is difficult to these results with our results.

TFS reflecting parenchymal destruction in IPF is associated with mortality. In our study, no correlation was found between US findings and TFS. In the study of He et al. on IPF, COPD, and CPFE patients, they found a negative significant correlation between diaphragmatic excursion during deep breathing and emphysema score. However, they found no correlation with the fibrosis score ( 19 ). When we divided our patients into two groups, TFS≤ 320 and TFS> 320 according to the median TFS value, we did not detect any difference between the ultrasonography findings. Differences between fibrosis score measurement methods make it difficult to compare these studies. Similarly, no study in the literature focuses particularly on IPF cases.

There are some limitations of our study. Firstly, the small sample size may raise concerns. In addition, while pulmonary function tests were applied to the patients in the sitting position, US was performed in the supine position; this may affect the correlation of measurements with functional parameters.

The available evidence related to respiratory muscle dysfunction in ILD is inconclusive. Therefore, some facts should be taken into account when interpreting muscle function. The ILD population included in the studies was heterogeneous in terms of age, gender, functional status, disease duration, and more importantly, the underlying pathology. All of these are potential confounding factors in the assessment of muscle strength. Furthermore, the findings/results obtained from studies conducted in small mixed patient groups cannot be generalized to the large ILD population. All this evidence regarding muscle dysfunction in IPF calls for further studies.

## CONCLUSION

Although it has the potential to predict pulmonary functions, the findings of this study do not support the use of diaphragmatic function evaluation for this purpose in early-stage disease. In our study, there were patients with mild to moderate restriction, but there were no patients who had severe restriction. In the later stages of the disease, insufficient compensatory response to the load on the respiratory muscles as a result of increased elastic recoil may lead to impaired diaphragmatic functions. To understand the relationships between respiratory muscle abnormalities, functional parameters, exercise capacity, quality of life, and mortality, additional research is required, including research on the advanced stages of the disease.

## Ethical Committee Approval

Ethics committee approval was obtained from Haydarpaşa Numune Training and Research Hospital Clinical Ethics Committee (Decision no: HNEAH-KAEK 2020/34- 2123, Date: 23.03.2020).

## AUTHORSHIP CONTRIBUTIONS

Concept/Design: All of authors

Analysis/Interpretation: All of authors

Data acqusition: All of authors

Writing: GKK, OO, ÖA

Clinical Revision: OO, ZK, CS, MY

Final Approval: OO, ZK
